# Transport and Optical Gaps in Amorphous Organic Molecular Materials

**DOI:** 10.3390/molecules24030609

**Published:** 2019-02-09

**Authors:** Emilio San-Fabián, Enrique Louis, María A. Díaz-García, Guillermo Chiappe, José A. Vergés

**Affiliations:** 1 Departamento de Química Física, Universidad de Alicante, 03080 Alicante, Spain; 2 Departamento de Física Aplicada, Universidad de Alicante, 03080 Alicante, Spain; enrique.louis@ua.es (E.L.); maria.diaz@ua.es (M.A.D.-G.); chiappe@ua.es (G.C.); 3 Departamento de Teoría y Simulación de Materiales, Instituto de Ciencia de Materiales de Madrid (CSIC), Cantoblanco, 28049 Madrid, Spain;jav@icmm.csic.es

**Keywords:** transport gap, optical gap, OLED, TD-DFT

## Abstract

The standard procedure to identify the hole- or electron-acceptor character of amorphous organic materials used in OLEDs is to look at the values of a pair of basic parameters, namely, the ionization potential (IP) and the electron affinity (EA). Recently, using published experimental data, the present authors showed that only IP matters, i.e., materials with IP > 5.7 (<5.7) showing electron (hole) acceptor character. Only three materials fail to obey this rule. This work reports ab initio calculations of IP and EA of those materials plus two materials that behave according to that rule, following a route which describes the organic material by means of a single molecule embedded in a polarizable continuum medium (PCM) characterized by a dielectric constant ε. PCM allows to approximately describe the extended character of the system. This “compound” system was treated within density functional theory (DFT) using several combinations of the functional/basis set. In the preset work ε was derived by assuming Koopmans’ theorem to hold. Optimal ε values are in the range 4.4–5.0, close to what is expected for this material family. It was assumed that the optical gap corresponds to the excited state with a large oscillator strength among those with the lowest energies, calculated with time-dependent DFT. Calculated exciton energies were in the range 0.76–1.06 eV, and optical gaps varied from 3.37 up to 4.50 eV. The results are compared with experimental data.

## 1. Introduction

The standard procedure to identify the hole- or electron-acceptor character of amorphous organic materials (AOM) is to look at the values of a pair of basic parameters, namely, the ionization potential (IP) and the electron affinity (EA). It is then widely accepted that those having high IP, EA will mainly act as an electron-acceptor, whereas those with low IP, EA are expected to behave as hole-acceptors [1,2], albeit, this rule of thumb is based upon rather solid grounds, its key drawback is a technical one; while IP is easily measured by means of direct ultraviolet photoelectron spectroscopy (UPS), EA requires inverse UPS, a technique not that simple and/or accessible. These difficulties force many researchers to derive EA indirectly through the measurement of the optical gap, a procedure that may introduce an error as high as 1 eV (actually, the exciton binding energy) in organic materials. Recently, using previously published experimental data [1], the present authors showed that, actually, only the ionization potential matters to identify the hole- or electron-acceptor character [3]. Data of IP were plotted against EA for up to 50 AOM (25 electron-acceptors and 25 hole-acceptors). Such a plot indicated that, except for three molecules, all others are separated into two groups: those having an IP > 5.7 eV corresponding to electron-acceptors, while hole-acceptors are confined to the region IP<5.7 eV. Moreover, density functional theory (DFT)/polarizable continuum medium (PCM) calculations with ε=4 for all molecules and a single functional/basis set combination, displace those three molecules to their respective correct region. These results indicate that, apparently, the EA plays a minor role, if any, in defining the two groups of charge transporter AOM.

In order to provide further support to the proposal of reference [3] we have undertaken the calculation of IP, EA and the exciton’s binding energy [4,5], and thus, the transport $G_{trans}$ and the optical gaps $G_{opt}$ (see below) by means of DFT/PCM, or time-dependant (TD)-DFT/PCM in the calculations of the exciton’s energy, of the three materials that fail to have the IP corresponding to their hole or electron character, plus two additional materials that do actually lie in their respective correct regions. Albeit there are alternative methods for the calculation of excited states, in particular we mention here the perturbative method GW [6,7,8,9,10] and the combination of these formalisms with a discrete polarizable model of atomistic resolution [11,12], methods similar to the approach adopted here are still used by many authors [4,5]. The extended character of AOM was taken into account incorporating a polarized continuum model [13] (the integral equation formalism variant of PCM used in the Gaussian09 [14] package). PCM are characterized by a dielectric constant ε that in most cases is only known to vary in a given range [15]. Thus, in the present calculations it was derived by requiring the fulfilment of Koopmans’ theorem. The optical gap was assumed to be determined by the excited state with the largest oscillator strength among those with the lowest energies. This methodology has several interesting aspects; (i) it provides a method to calculate the dielectric constant in cases where experimental data are lacking or not sufficiently precise and/or trustable, (ii) investigate how the different combinations of functional/basis sets affect the fulfillment of Koopmans’ theorem and, thus, the calculation of the dielectric constant, and (iii) provide a route for the calculation of IP, EA and transport and optical gaps of AOM.

At this point it is worth precisely defining some magnitudes used throughout this paper. The so called “fundamental gap” is given by the difference of the ionization potential and the electron affinity, i.e., G = IP − EA, which in turn are defined as IP = $E^{+}-E^{0}$ and EA = $E^{0}-E^{-}$, where $E^{0}$ is the ground state energy of the neutral molecule, while $E^{\pm }$, are the molecule ground states with plus or minus one electron. Both are usually calculated with the geometry of the neutral molecule (assumption that is supported by the very different time scales of vibrons and electrons). When dealing with extended systems the fundamental gap is renamed as “band gap”, or more graphically as the “transport gap” as it amounts to the minimum energy required to create mobile charges either in the conduction band (electrons) and/or in the valence band (holes). In many cases the fundamental gap is identified with $E_{HOMO}-E_{LUMO}$. However, this is only an approximation that, in cases, can be very rude, as the latter difference can significantly depend on the details of the specific methodology used in their calculations. Finally, the “optical gap” ($G_{opt}$) defined as the energy of the lowest electronic transition promoted by absorption of a single photon. The exciton binding energy is thus given by $E_{exciton}=G_{opt}-G_{trans}$. This binding energy is very small in inorganic compounds (of the order of meV) and larger in organic materials (around one eV). This large difference cannot be solely ascribed to the dielectric constant (ε), which is at most 20 in inorganic systems and 3–5 in most organic materials, but rather it is surely due to differences in many-body interactions (see below). A more detailed discussion of these issues can be found in references [2,3].

The paper is organized as follows. The first section deals with the methods and numerical procedures, starting with a subsection devoted to comment general aspects of the theoretical and computational framework followed here to calculate gaps and exciton binding energies. Specific features of our methods are described in the next subsection. Results of our calculations are discussed thereafter and compared to available experimental data. The last section is devoted to comment on the main conclusions of our work.

## 2. Methods and Numerical Computations

### 2.1. General Considerations

The optical transition with the lowest energy and a significant oscillator strength, produced by photon absorption in molecules or materials, defines the optical gap. This transition creates a bound electron-hole pair more or less localized depending on the characteristics of the molecule or material. The electron-hole pair can be considered as a quasiparticle, usually called exciton, as it can move from molecule to molecule. The difference between the ground state and the lowest excited state (see below), or, equivalently, between the transport gap and the optical gap, defines the exciton binding energy $E_{exciton}$. A standard method to calculate the exciton binding energy $E_{exciton}$ of a molecule consists of: (i) computing the transport gap $G_{trans}$ defined as the difference IP-EA (see above) or, alternatively, one may derive both magnitudes from the results of the DFT calculation for the highest occupied molecular orbital (HOMO) and lowest occupied molecular orbital (LUMO) levels and, assuming Koopmans’ theorem to be valid (see below), write IP = $-E_{HOMO}$ and EA = $-E_{LUMO}$, (ii) calculating the excited states by means of a suitable method (see next section) from which, by taking into account the oscillator strengths, the optical gap $G_{opt}$ is derived, and, as already pointed out, the exciton binding energy is given by $E_{exciton}=G_{opt}-G_{trans}$.

Koopmans’ theorem [16] states that in closed-shell Hartree–Fock (HF), the first ionization energy of a molecular system is equal to the energy of the HOMO, with its sign changed. The main sources of error are well known: (a) the lack of orbital relaxation; and (b) the fact that, being HF a single determinant method, the electronic correlation is poorly described. A similar theorem exists in density functional theory (Janak’s theorem [17]) for relating the first vertical ionization energy and the electron affinity to the HOMO and LUMO energies, respectively, obtained with an exact exchange-correlated density functional [17,18,19,20,21,22]. The error in the DFT counterpart of Koopmans’ theorem is a result of the approximation employed for the exchange-correlation energy functional so that, unlike in HF theory, it is possible to improve the results with the development of better approximations or, particularly, better functionals. At this point, it is worth noting that when an electron is removed or added to an extended systems (as the AOM here studied), both orbital relaxation and electron correlation decrease significantly with respect to a localized media, and thus, one may expect IP = $-E_{HOMO}$ and EA = $-E_{LUMO}$ to be a reasonable approximation.

In the present case the extended character of AOM was introduced using a polarized continuum model (PCM) [13], which has been, and still is being, profusely utilized by many authors [23,24,25,26]. Incorporating PCM into traditional DFT amounts to actually solving a “compound system” formed by a single molecule immersed into a continuum polarizable medium. Although recent efforts to incorporate solid state effects more accurately have been made [27,28,29], it is obvious that a continuum model is more appropriate to describe amorphous solids [30]. The PCM is characterized by a dielectric constant ε (the ratio of the permittivity of a substance to the permittivity of free space). Calculations were done for a dielectric constant varying in the range 1–80 (ε = 1 corresponds to a single molecule in vacuum). However, as the dielectric constant of molecular materials is known to vary in the range 3–6 [2,30,31,32,33]. Results for ε in the range 1–10 are only displayed in the Figures. The optimal value of ε is that at which the curves IP(EA)-vs.-ε and $E_{HOMO}$ (LUMO)-vs.-ε cross, or in other words, that at which Koopmans’ theorem holds.

Several published works take the dielectric constant from either experimental data or approximate expressions and, subsequently, attempt to optimize the exchange and correlation functional by requiring the fulfillment of Koopmans’ theorem (see, for instance, [34,35,36,37]). In particular, some authors seek a relation between the amount of HF exchange included in their calculations and the dielectric constant [24]. We believe that our approach is more transparent as it is only based upon Koopmans’ theorem and the dielectric constant, whereas that followed in [24,34,35,36,37] incorporates changes in the functional.

### 2.2. Specific Procedures

DFT calculations were carried out using six functionals, two generalized gradient approximation (GGA) exchange-correlation functionals, BLYP [38,39] and PBEPBE [40,41], two hybrid functionals, B3LYP [42,43] and PBE0 [44,45] and one meta-GGA functional, τHCTH [46] with three basis sets, Def2SV, Def2TZV and Def2TZVPP [47,48]. As some of the excited states can be of global electron density transfer (GEDT) nature, we have checked the performance of a few long range corrected functionals (for instance CAM-B3LYP [49] and LC-ωPBE [50]). However, as these functionals reproduce Koopmans’ theorem for ε close to 1 [51], well below that expected for the molecules studied here, we have discarded all long range functionals. This does not imply that these functionals can be used for the calculation of gaps in solids, for which the relationship of the long-range exact exchange has been found with the dielectric constant (See reference [52] and their references). Calculations have been made for the different functionals with each basis set and varying the values of the dielectric constant used in the PCM. In all cases the geometry of the neutral ground state has been optimized.

According to the present analysis, the basis set may vary the value of ε at which the curves IP(EA)-vs.-ε and $E_{HOMO}$ (LUMO)-vs.-ε cross for a given molecule, albeit, crossing occurs in any case. However, not all functionals lead to such a crossing (see below).

Finally, using the PBE0/Def2TZVPP, vertical excitation energies have been calculated with TD-DFT [53,54,55,56] method. The dielectric constant ε used in these calculations was the average of those for which IP = $-E_{HOMO}$, or EA = $-E_{LUMO}$ (both are usually very similar). A self-consistent evaluation of the effects of solvent on the excited state has been made. The linear response formalism [57,58] was used to calculate the ground state optimized geometry (frequencies have been calculated to confirm its nature), and the vertical excitation was estimated with a non-equilibrium solvation linear response calculation. Finally, for the chosen excited states, the effects of solvation have been improved with a state-specific calculation [59,60,61]. All calculations were done with the Gaussian09 package [14], using Jmol [62], Gabedit [63] or Molden [64] for displaying and analyzing the results.

## 3. Results

### 3.1. Transport Gap

**Figure 1:**
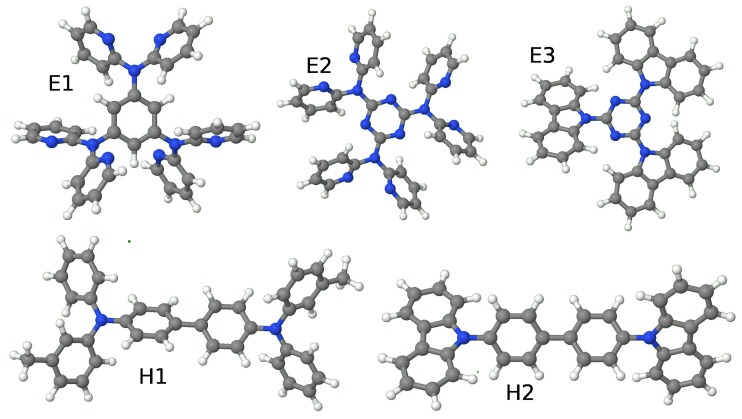
Schematic representation of the systems here studied, three electron-acceptors: 1,3,5-tris(phenyl-2-pyridylamino)benzene (E1) 2,4,6-tris[di(2-pyridyl)-amino]-1,3,5-triazine (E2) and 2,4,6-tris(carbazolo)-1,3,5-triazine (E3) plus two hole-acceptors: N,N′-Bis(3-methylphenyl)-N,N′-diphenylbenzidine (H1) and 4,4′-di(N-carbazolyl)biphenyl (H2).

Calculations have been carried out for the systems studied in the previous paper (See reference [3]), three electron-acceptors: 1,3,5-tris(phenyl-2-pyridylamino)benzene (E1), 2,4,6-tris[di(2-pyridyl)-amino1,3,5-triazine (E2) and 2,4,6-tris(carbazolo)-1,3,5-triazine (TRZ2) (E3), (with numbers 40, 169 and 170, respectively, as assigned in reference [1]) and two hole-acceptors: N,N′-Bis(3-methylphenyl)-N,N′-diphenylbenzidine (TPD) (H1) and 4,4′-di(N-carbazolyl)biphenyl (CBP) (H2) (molecules 86 and 91 in reference [1]). (See Figure 1). Hereafter we first consider how the optimal dielectric constant was determined and, subsequently, the transport gap.

**Figure 2:**
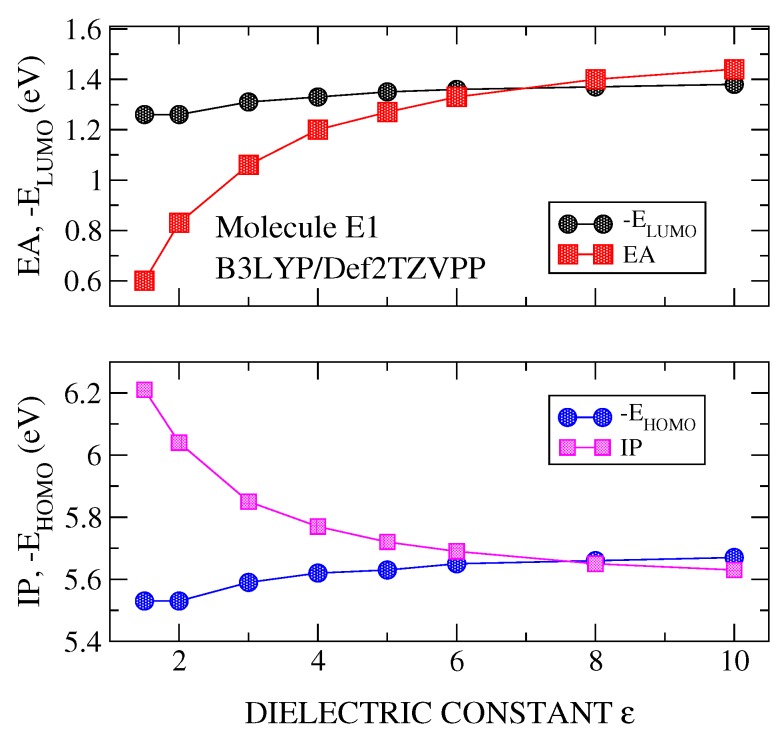
Calculated values of ionization potential (IP) and $E_{HOMO}$ (**lower panel**) and electron affinity (EA) and $E_{LUMO}$ (**upper panel**) for molecule E1. Calculations were carried out with B3LYP/Def2TZVPP method and using polarizable continuum medium (PCM) with a dielectric constant ε varying in the range 1–10.

**Figure 3:**
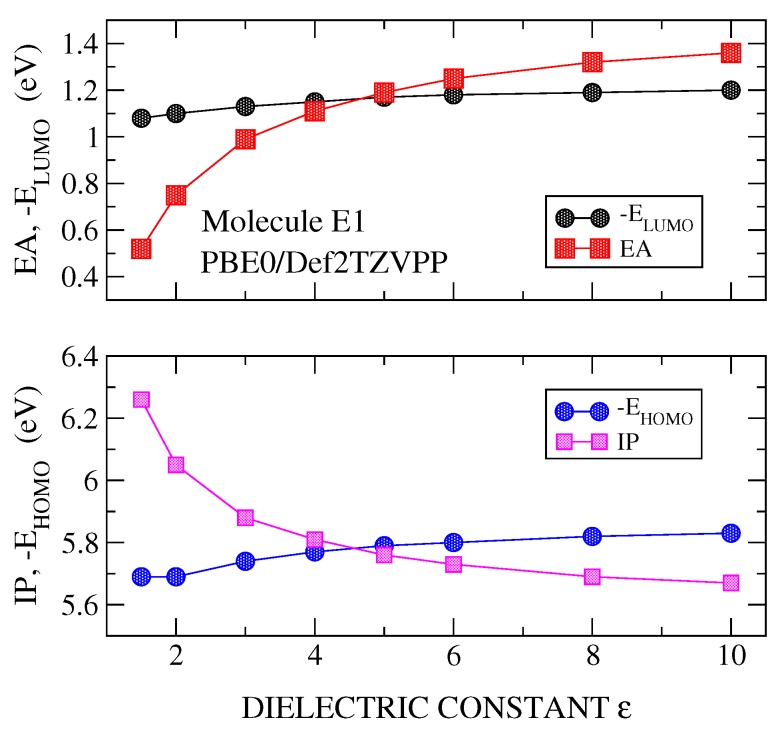
Calculated values of IP and $E_{HOMO}$ (**lower panel**) and EA and $E_{LUMO}$ (**upper panel**) for molecule E1. Calculations were carried out with PBE0/Def2TZVPP method and using PCM with a dielectric constant ε varying in the range 1–10.

**Figure 4:**
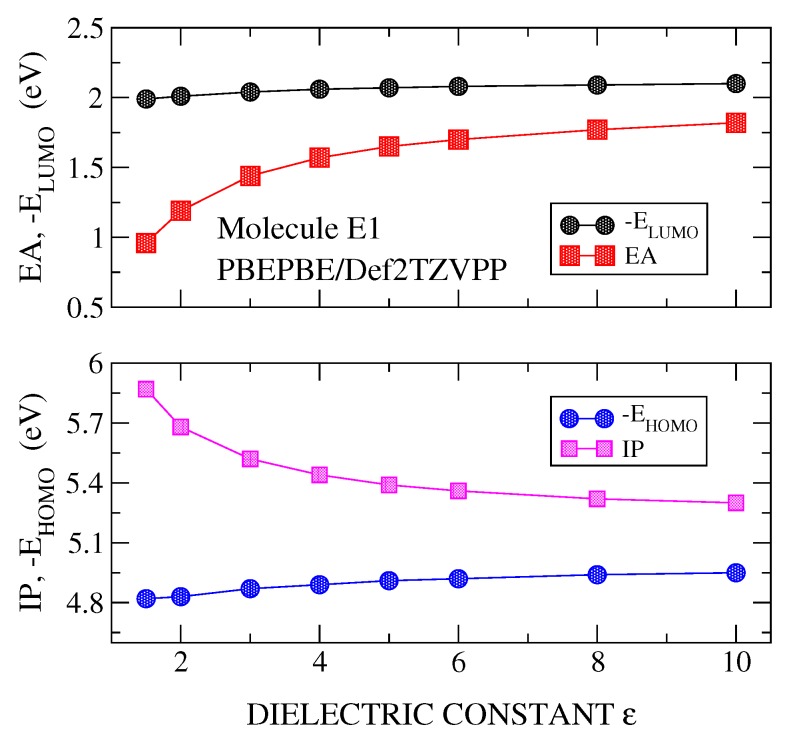
Calculated values of IP and $E_{HOMO}$ (**lower panel**) and EA and $E_{LUMO}$ (**upper panel**) for molecule E1. Calculations were carried out with PBEPBE/Def2TZVPP method and using PCM with a dielectric constant ε varying in the range 1–10.

DFT plus PCM calculations were carried out for the IP, EA, $E_{HOMO}$ and $E_{LUMO}$, versus the dielectric constant (ε). Figure 2, Figure 3 and Figure 4 show the results for the molecule E1, although similar results are obtained for the rest of the studied molecules. It is noted that the polarizable character of PCM is manifested clearly in the stronger effects it has on the parameters of the charged system (IP and EA) than on those of the neutral system ($E_{HOMO}$ and $E_{LUMO}$). These calculations have been made using the best basis set (Def2TZVPP) and three functionals, B3LYP (Figure 2), PBE0 (Figure 3) and PBEPBE (Figure 4). The first two hybrid functionals show a crossing of the IP-vs-ε and $E_{HOMO}$-vs.-ε curves, and, the EA-vs.-ε and $E_{LUMO}$-vs.-ε curves. The crossing of the IP-$E_{HOMO}$ and EA-$E_{LUMO}$ curves occurred at very similar values of the dielectric constant, namely, ε=7.8 and ε=7.2 for B3LYP and ε=4.7 and at ε=4.6 for PBE0 (see Figure 2 and Figure 3 and Table 1). Although both functionals show crossing, thus allowing us to derive an optimal dielectric constant from this analysis, we chose PBE0 for the majority of the calculations presented here for two reasons; most theoretical analyses support PBE0 as the most suitable functional for organic materials and molecules [4], and the dielectric constant derived from the use of this functional gives ε values well within the range 3–6, which is the expected range for the materials at hand [2]. Note that the rather popular B3LYP functional gives dielectric constants as high as 9.4 (see molecule E2 in Table 1). On the other hand, with the PBEPBE functional (Figure 4) no crossing was found over a rather wide range (1–80). In fact, this is not the only functional that did not show crossing, rather at least two additional functionals were identified (BLYP and τHCTH). For the long range corrected functionals, these crossing occur at values close to ε=1, as it was indicated previously.

The qualitative effect that the choice of the functional may have had on the results for the optimal dielectric constant was not seen when the basis set was changed. The use of a different basis set may have varied the values of ε at which the curves IP(EA)-vs.-ε and $E_{HOMO}$ (LUMO)-vs.-ε cross, albeit for the molecules investigated here crossing occured no matter the basis. This is illustrated by the results reported in Table 1 for the optimal dielectric constant, obtained with the PBE0 functional and the three previously indicated basis sets. It is noted that the maximum difference was always smaller than 15%, being in most cases smaller than 5%. In the following, the dielectric constant introduced in the PCM will be the average of those at which the curves IP(EA)-vs.-ε and $E_{HOMO}$ (LUMO)-vs.-ε, obtained with the combination PBE0/Def2TZVPP.

### 3.2. Optical Gap and Exciton Binding Energies

Energies of the excited states for each of the five organic molecular materials investigated here were calculated within TD-DFT framework with the hybrid exchange-correlation functional PBE0 and using the Def2TZVPP basis set and with a procedure similar to that of reference [4]. The dielectric constant was calculated as explained in the previous subsection and incorporated into the PCM. The calculated energies for the four lowest lying excited states are reported in Table 2. Their oscillator strengths are also given. It is noted that while the energies of the four excited states of each molecule did not differ much, their oscillator strengths may have been significantly different, determining, for molecules H1 and H2 (hole-acceptors) the main transition and thus, the first excited state. Nevertheless, for molecules E1, E2 and E3 (electron-acceptors), the first excited state was not that one which shows the larger oscillator strength. Then, for the first excited state of H1 and H2 together with the first three excited states of the systems E1, E2 and E3, state-specific (SS) solvation calculations were carried out, within the non-equilibrium solvation-linear response approximation (See references [13,65]). The results for the most important implicated excitations and their oscillator strengths are shown in Table S6 of the electronic supporting information (ESI). A summary which includes the results of the excited states of less energy having a considerable oscillator strength is shown in Table 3. As expected, solvation calculations slightly decreased the energy of the excited states, and significantly modified the value of the oscillator strengths.

Table 4 reports experimental data collected in reference [1] for the IP, EA and the optical gap (all in eV). Theoretical results (last five columns) were obtained by means of PBE0/Def2TZVPP and incorporating the PCM with the dielectric constant ε derived as explained succinctly in Table 1 and extensively in the main text. $G_{trans}$ and $G_{opt}$ stand for transport and optical gap, respectively. It is noted that theoretical results for $G_{opt}$, albeit slightly larger than the experimental data, are not that different. Some hints that may help to understand this discrepancy are outlined in the following paragraph. As regards the exciton binding energies, they are of the order of the expected values for these materials. However, it is likely that due to overestimation of $G_{opt}$, our calculations underestimated their actual values. As remarked above, solvation calculations reduce in a small amount (less than 0.2 eV) the excited state energy and, thus, the optical gap.

Exciton binding energies in organic materials are usually much larger than those currently observed in inorganic compounds, namely, i.e., meV, as compared to tenths of eV typical of organic materials. However, it is unlikely that this large difference in $E_{exciton}$ may be solely understood in terms of differences in the dielectric constants. Instead, it is widely accepted that electron-electron and electron-phonon interactions may also play a substantial role. Both interactions are commonly larger in inorganic materials, contributing appreciably to the reduction of the optical gap and, thus, the exciton binding energy. The no-consideration of electron-phonon interactions in the present calculations, the incomplete treatment of electron-electron interactions, and the different nature of their electronic transition may surely be the reasons of the difference in the results showed above.

**Figure 5:**
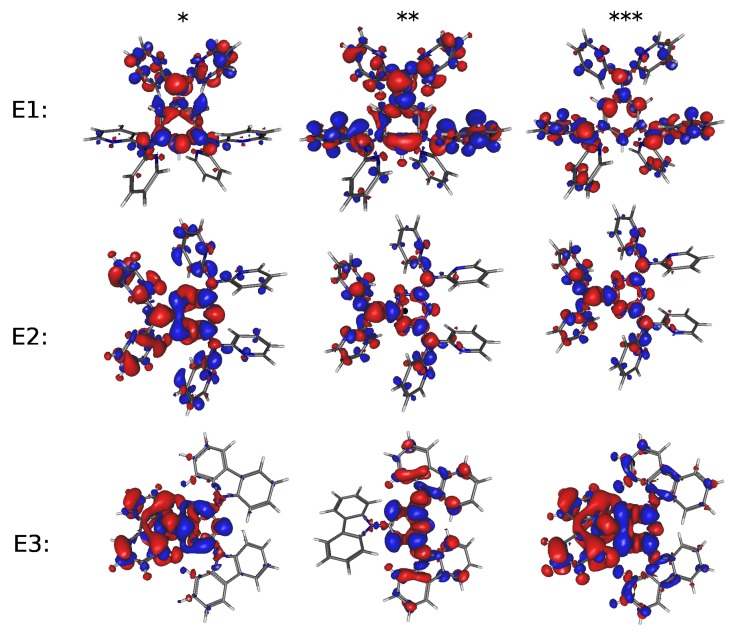
Iso-contour plots (cutoff value of 0.001 au.) of ground- and excited-state density difference for the electron-acceptor systems here studied (E1, E2 and E3). *, ** and *** refer to the first, second and third excited-states, respectively.

**Figure 6:**
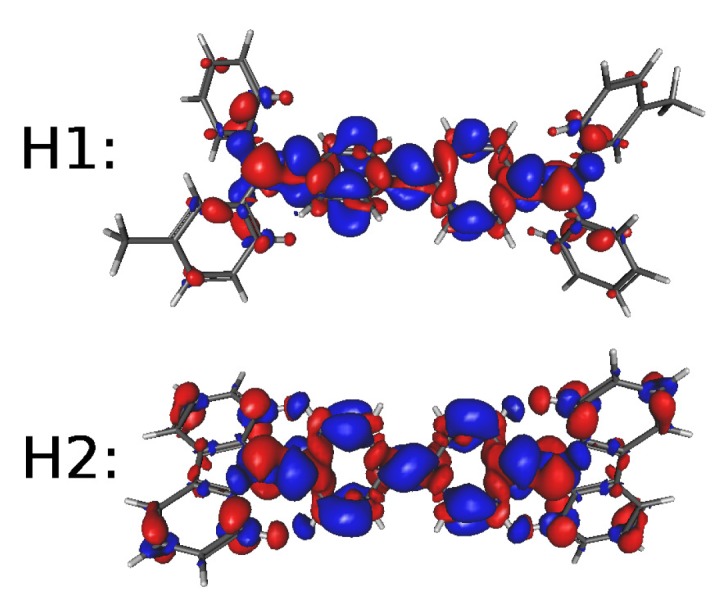
Iso-contour plots (cutoff value of 0.001 au.) of ground- and the first excited-state density difference for the hole-acceptor systems H1 and H2.

In order to attain an understanding of the nature of the electronic transitions in these systems, it is worth drawing iso-contour plots of the difference between the local electron densities in ground- and excited-states (Figure 5 and Figure 6). Thus, calculations of the GEDT between the ground- and the excited-states have been carried out using the procedure of Le Bahers et al. [66]. Table 5 shows the results (see full results in Table S7 of ESI) for the charge transferred (CT): $q^{CT}\;=\;\int \rho_{+}\left(r\right)\;dr\;=\;\int \rho_{-}\left(r\right)\;dr$, r being the position vector in three-dimensions. In this expression $\rho_{+}\left(r\right)$ and $\rho_{-}\left(r\right)$ stand for positive or negative differences between densities of the excited and ground state wave-functions that can be quantified by the spatial distance between the barycenters of these density distributions $d^{CT}=\left|\frac{\int r\rho_{+}\left(r\right)dr}{\int \rho_{+}\left(r\right)dr}-\frac{\int r\rho_{-}\left(r\right)dr}{\int \rho_{-}\left(r\right)dr}\right|$ and the difference between the dipole moments computed for the ground and the excited states $\mu^{CT}=d^{CT}q^{CT}$.

It is readily noted in Figure 6 that, in hole-acceptor systems, the excitation localizes on the central part of the molecule backbone (see reference [4]) and the spatial distance between the barycenters of the two density distributions $d^{CT}$ has values that are far from the range within which GEDT excitations usually lie (≥1.5 Å) Then, they should be considered as local excitations. The three electron-acceptor systems E1 and E2, in turn, show a second and a third excited state with considerably higher oscillator strengths, such that, albeit having higher energies, may compete with the first excited state. Actually, the very small oscillator strength of the latter makes more probable the excitation to the second excited state.

A consequence of what has been pointed out in the preceding paragraph is that the excited states of systems E1 and E3 can be safely considered as GEDT excited states, with $d^{CT}$ approximately equal to 1.95 and 2.40 Å. Instead, material E2, albeit having a first excited state with $d^{CT}$ of 2.25 Å, its small oscillator strength indicates that the intensity of the excitation might be small compared to the transition to the second excited state, that, with $d^{CT}$ of only 0.57 Å, should be considered as a local excitation. These remarks are better illustrated by Figure 5 that shows iso-contour plots of ground- and excited-state density difference for the three electron-acceptor systems. Figures S1–S5 of the ESI show iso-contour plots of some of the molecular orbitals likely involved in the transitions that define the optical gap.

## 4. Concluding Remarks

The aim of the present work was to calculate the optical gap and exciton binding energies of five AOM used frequently in the fabrication of OLEDs, by means of methods of quantum chemistry commonly used for these purposes. The minimal requirements that a method should have to deal with this system with a reasonable probability of success are the following:

- Calculation of the ground state wave-function and the excitation energy within time independent DFT framework and using high performance combinations of a functional/basis set.
- Incorporate the extended character of the system implemented in the present work by means of a polarized continuum model.
- Find a way to determine the dielectric constant, the key parameter of PCM. Measurements or estimation with simplified models are commonly used [1,30,67]. Here we have adopted a different approach that allowed to obtain an optimal value of ε imposing the fulfillment of Koopmans’ theorem.
- Our results reveal the importance of considering the exact exchange energy in the calculation of the optimal value of ε.

Altogether, we believe our procedures to be a feasible path to follow in handling a system as complicated as the one here studied. Hereafter, we summarize the main results. Calculations were carried out for five AOM used in OLEDs either as electron- (three of them) or hole-acceptors. Optimal dielectric constants varied in the range 4.4–5.0, exciton energies from 0.71 up to 1.06 eV, and optical gaps from 3.37 up to 4.50 eV.

The latter were in all cases larger than experimental data in amounts that vary from 0.2 to 0.8 eV, probably due to several factors, namely, an incomplete treatment of the electron-electron interaction, the use of polarized continuum models, experimental errors, etc.

A direct exciton energy calculation requires the calculation of the two-particle spectrum by solving the Bethe–Salpeter equation. The development of time-dependent DFT has paved the road [68]. Thus, albeit the discussion concerning the suitability of TD-DFT for a proper description of excitonic matter is still alive, we have followed this approach as a pragmatic choice. It is pertinent to mention here that in a very recent work [69] it has been shown how to merge the Bethe–Salpeter equation with the PCM formalism.

Finally, it is worth noting that the present results not only do not contradict the analysis of our previous work [3], in which calculations were carried out with ε=4 for all molecules, while in the present case a different ε was used for each molecule, (see Table 1). But rather, in all cases but one, they reinforce our conclusions concerning the character of AOM, namely, those with IP >5.7 expected to be electron-acceptors, while those having IP <5.7 predicted to be hole-acceptors. Specifically, for the electron-acceptor materials E1, E2 and E3, the present calculations give IP values of 5.77, 6.56 and 6.37 eV, respectively, while in our previous work we reported 5.77, 6.49 and 6.3 eV, respectively. In turn, in the case of hole-acceptors, the IP increases in both molecules albeit slightly, namely, for H1 we previously reported 5.19 eV, versus a value of 5.23 eV in the present work. For molecule H2 here we obtained 5.87 eV, quite close to the value reported previously 5.83 eV [3] (both near the critical value of 5.7 eV).

## Figures and Tables

**Table 1:** Ionization potential (IP) and electron affinity (EA) both in eV, of three electron-acceptor (E1, E2 and E3) and two hole-acceptors (H1 and H2) in amorphous organic materials (AOM) calculated using DFT with four functional/basis-set combinations, and polarizable continuum medium (PCM). The reported dielectric constants ε (used also in the calculations of IP and EA) are those at which IP = $-E_{HOMO}$, or EA = $-E_{LUMO}$ (see Figure 2 and Figure 3).

**Molecule**	**E1**		**E2**		**E3**		**H1**		**H2**	
**Functional/Basis Set**	ε	**IP**	ε	**IP**	ε	**IP**	ε	**IP**	ε	**IP**
B3LYP/Def2TZVPP	7.8	5.66	9.4	6.40	9.0	6.14	8.0	5.07	8.0	5.70
PBE0/Def2TZVPP	4.6	5.78	4.8	6.55	5.0	6.36	4.7	5.22	4.8	5.86
PBE0/Def2TZV	4.7	5.81	4.8	6.55	5.2	6.39	4.8	5.22	5.0	5.88
PBE0/Def2SV	4.9	5.79	4.8	6.55	5.5	6.39	5.0	5.25	5.2	5.91
**Molecule**	**E1**		**E2**		**E3**		**H1**		**H2**	
**Functional/Basis Set**	ε	**EA**	ε	**EA**	ε	**EA**	ε	**EA**	ε	**EA**
B3LYP/Def2TZVPP	7.2	1.36	8.1	1.37	8.2	1.78	8.1	1.28	8.4	1.52
PBE0/Def2TZVPP	4.7	1.16	4.8	1.13	4.4	1.59	4.6	1.12	4.9	1.38
PBE0/Def2TZV	4.7	1.10	4.8	1.19	4.8	1.69	4.8	1.02	5.2	1.29
PBE0/Def2SV	5.0	1.08	5.1	1.12	5.0	1.64	5.0	1.12	5.6	1.43

**Table 2:** Calculated energies of the lowest excited states $E_{exc-sta}$, and their respective oscillator strengths, in the AOM investigated in this work. Calculations were carried out within the time-dependent density functional theory (DFT) framework using the combination PBE0/Def2TZVPP and polarizable continuum medium (PCM) with the dielectric constant ε derived as explained in the main text (see also Table 1).

No.	Molecule	$E_{\mathrm{exc}-\mathrm{sta}}$ (eV)	Oscillator Strength
**E1**	1,3,5-tris(phenyl-2-pyridylamino)benzene	3.745	0.024
		3.891	0.094
		3.944	0.211
		4.026	0.023
**E2**	2,4,6-tris[di(2-pyridyl)-amino]-1,3,5-triazine	4.480	0.207
		4.511	0.352
		4.525	0.226
		4.646	0.092
**E3**	2,4,6-tris(carbazolo)-1,3,5-triazine (TRZ2)	3.911	0.326
		4.023	0.011
		4.040	0.421
		4.149	0.015
**H1**	N,N′-Bis(3-methylphenyl)-N,N′-diphenylbenzidine (TPD)	3.405	1.122
		3.655	0.000
		3.738	0.025
		3.910	0.001
**H2**	4,4′-di(N-carbazolyl)biphenyl (CBP)	3.772	0.714
		3.974	0.000
		3.999	0.093
		4.018	0.000

**Table 3:** Calculated energies (in eV) of the lowest excited states with a large oscillator strengths (>0.1), in the amorphous materials based on the five organic molecules investigated in this work. Calculations were carried out within the time-dependent DFT framework using the combination PBE0/Def2TZVPP and a state-specific solvation calculation, with the dielectric constant ε derived as explained in the main text and reported in Table 1.

Molecule	Excited State	$E_{\mathrm{exc}-\mathrm{sta}}$ (eV)	Oscillator Strength
**E1**	2	3.807	0.116
**E2**	2	4.505	0.354
**E3**	1	3.752	0.301
**H1**	1	3.373	1.109
**H2**	1	3.604	0.632

**Table 4:** Experimental data (superscript exp) collected in reference [1] for the IP, EA, and optical gap (all in eV) of the five AOM investigated in this work. Theoretical results (last five columns) were obtained by means of DFT with the combination PBE0/Def2TZVPP and using PCM with the dielectric constant ε derived as explained extensively in the main text. $G_{trans}$ and $G_{opt}$ stand for transport and optical gap, respectively. The expression $EA=IP^{exp}-G_{opt}^{exp}$ is only exact when the optical and transport gap coincide, it i.e., the exciton binding energy is null (see beginning of Section 1).

Molecule	IP${}^{\exp}$	$G_{\mathrm{opt}}^{\exp}$	$\mathrm{EA}={\mathrm{IP}}^{\exp}-G_{\mathrm{opt}}^{\exp}$	ε	IP	EA	$G_{\mathrm{trans}}$	$G_{\mathrm{opt}}$	$E_{\mathrm{exciton}}$
**E1**	5.09	3.45	1.64	4.62	5.78	1.16	4.62	3.81	0.81
**E2**	5.07	3.72	1.35	4.80	6.56	1.14	5.42	4.50	0.92
**E3**	6.0	3.4	2.60	4.72	6.37	1.56	4.81	3.75	1.06
**H1**	5.50	3.2	2.3	4.65	5.23	1.13	4.1	3.37	0.73
**H2**	6.3	3.1	3.2	4.88	5.87	1.39	4.48	3.60	0.88

**Table 5:** Charge-transfer parameters computed using the density for the main excited state (second column) of each molecule here considered (first column). Units used for the calculated magnitudes are given in parenthesis.

Molecule	Excited State	$q^{\mathrm{CT}}$ (Electron-Charge)	$d^{\mathrm{CT}}$ (Å)	$\mu^{\mathrm{CT}}$ (Debye)
**E1**	2	0.768	1.915	7.068
**E2**	2	0.472	0.571	1.293
**E3**	1	0.832	2.400	9.597
**H1**	1	0.572	0.396	1.088
**H2**	1	0.857	0.014	0.056

## Supplementary Materials

The Supplementary Materials are available online.

## Abbreviations

The following abbreviations are used in this manuscript:

AOM	Amorphous organic materials
B3LYP	Hybrid BLYP functional
BLYP	Becke-Lee-Yang-Parr exchange correlation functional
CAM-B3LYP	Long-range-corrected version of B3LYP functional
CT	Charge transferred
DFT	Density functional theory
EA	Electron affinity
GEDT	Global electron density transfer
GGA	Generalized gradient approximation
G	Fundamental gap
G${}_{trans}$	Transport gap
G${}_{opt}$	Optical gap
HOMO	Highest occupied molecular orbital
IP	Ionization potential
LC-ωPBE	Long-range-corrected ωPBE functional
LUMO	Lowest unoccupied molecular orbital
OLED	Organic light-emitting diode
PCM	Polarizable continuum medium
PBEPBE	Perdew, Burke and Ernzerhof exchange correlation functional
PBE0	Hybrid PBEPBE functional
TD-DFT	Time-dependent DFT
τHCTH	The τ-dependent Handy’s functional
