# Supplementary material for: Transport and Optical Gaps in Amorphous Organic Molecular Materials
Source: Molecules. 2019 Feb 9;24(3):609. doi: 10.3390/molecules24030609 (PMC6384593; doi:10.3390/molecules24030609)
Supplement: Supplementary file 1 [file molecules-24-00609-s001.pdf]

Supplementary Information
Transport and optical gaps in Amorphous Organic Molecular Materials

Emilio San-Fabián*†‡ Enrique Louis§‡
María A. Díaz-García§‡ Guillermo Chiappe§‡
José A. Vergés¶‡

January 21, 2019

Contents

1 **Tables reporting for the five organic molecules investigated in this work:** **2**

2 **Graphical representations of several molecular orbitals of the five molecules here studied.** **10**

*corresponding author
†Departamento de Química Física, Universidad de Alicante, 03080 Alicante, Spain; E-mail: sanfa@ua.es
‡Unidad Asociada del CSIC and Instituto Universitario de Materiales, Universidad de Alicante, 03080 Alicante, Spain.
§Departamento de Física Aplicada, Universidad de Alicante, 03080 Alicante, Spain
¶Departamento de Teoría y Simulación de Materiales, Instituto de Ciencia de Materiales de Madrid (CSIC), Cantoblanco, 28049 Madrid, Spain.

1 Tables reporting for the five organic molecules investigated in this work:

- Ground state optimized geometries.
- The calculated energies of the lowest excited states and their respective oscillator strengths.
- The charge-transfer parameters computed using the local density of states.

Table S1: Optimized geometry of molecule **E1**, using PBE0/Def2TZVPP method.

Atomic	Coordinates (Angstroms)			Atomic	Coordinates (Angstroms)		
Number	X	Y	Z	Number	X	Y	Z
7	-4.671165	-1.273153	0.549484	6	5.808705	-0.581322	1.439336
7	-2.346626	-1.438668	0.638692	6	4.590733	-0.459695	2.096688
7	-1.455760	-2.128786	2.663207	6	3.509972	-1.154056	0.084082
7	1.466276	4.548377	-0.248525	6	-1.154286	-0.736194	0.319323
7	0.000234	2.760091	0.000024	6	-1.152520	0.651331	0.342992
7	-1.465486	4.548650	0.248507	6	0.000115	1.353932	0.000021
7	1.455364	-2.129054	-2.663170	6	1.152629	0.651137	-0.342953
7	2.346375	-1.439065	-0.638677	6	1.154159	-0.736389	-0.319297
7	4.670943	-1.273875	-0.549532	6	-0.000125	-1.436921	0.000009
6	-3.423676	-0.753126	-1.422935	1	-2.462630	-0.689332	-1.914414
6	-4.590768	-0.458982	-2.096734	1	-4.551362	-0.151133	-3.134910
6	-5.808775	-0.580446	-1.439416	1	-6.746117	-0.361780	-1.933224
6	-5.786046	-1.001041	-0.121883	1	-6.712175	-1.117704	0.433171
6	-3.510158	-1.153489	-0.084097	1	-3.832122	-3.674079	0.861040
6	-3.146233	-3.499906	1.676678	1	-3.671970	-5.276586	2.737474
6	-3.051066	-4.388633	2.725885	1	-2.041003	-4.819281	4.590881
6	-2.145592	-4.144204	3.751926	1	-0.641923	-2.771476	4.433277
6	-1.369705	-3.004160	3.661557	1	1.498653	2.182962	2.134953
6	-2.325704	-2.367145	1.689661	1	3.488630	3.485509	2.856974
6	1.824883	3.054186	1.583023	1	4.140658	5.518734	1.539788
6	2.926749	3.785242	1.980390	1	2.760767	6.128502	-0.433444
6	3.288166	4.914672	1.258681	1	-1.498267	2.183178	-2.134909
6	2.516724	5.252223	0.159723	1	-2.759693	6.129014	0.433371
6	1.123104	3.475456	0.452788	1	-4.139674	5.519442	-1.539858
6	-1.824344	3.054477	-1.583005	1	-3.488000	3.486065	-2.856985
6	-2.515801	5.252674	-0.159770	1	3.831472	-3.674745	-0.861009
6	-3.287294	4.915234	-1.258727	1	3.671013	-5.277250	-2.737419
6	-2.926074	3.785720	-1.980403	1	2.040105	-4.819681	-4.590813
6	-1.122500	3.475646	-0.452773	1	0.641389	-2.771627	-4.433219
6	3.145607	-3.500459	-1.676643	1	2.462558	-0.689754	1.914427
6	3.050268	-4.389186	-2.725836	1	6.711979	-1.118701	-0.433278
6	2.144827	-4.144611	-3.751869	1	6.746090	-0.362783	1.933116
6	1.369141	-3.004427	-3.661506	1	4.551398	-0.151840	3.134865
6	2.325282	-2.367551	-1.689635	1	-2.048320	1.182719	0.636169
6	3.423583	-0.753680	1.422921	1	2.048520	1.182372	-0.636129
6	5.785881	-1.001913	0.121803	1	-0.000218	-2.518721	0.000004

Table S2: Optimized geometry of molecule **E2**, using PBE0/Def2TZVPP method.

Atomic	Coordinates (Angstroms)			Atomic	Coordinates (Angstroms)		
Number	X	Y	Z	Number	X	Y	Z
7	-2.058279	4.110173	-0.701984	6	4.418463	3.655865	0.469888
6	-1.542353	3.282690	0.191906	6	4.024008	4.489010	-0.562145
6	-0.770318	3.703083	1.268727	6	3.077034	4.012374	-1.458119
6	-0.519061	5.054966	1.399125	6	2.568528	2.739598	-1.288548
6	-1.052793	5.935320	0.468107	6	-2.055970	-2.979991	0.202191
6	-1.820227	5.410969	-0.557077	6	-2.802962	-2.512801	1.278064
7	-1.869436	1.911027	0.009249	6	-4.087807	-2.994558	1.434399
6	-3.247546	1.613019	-0.175130	6	-4.576456	-3.928174	0.530887
6	-3.682690	0.853702	-1.254996	6	-3.742532	-4.337068	-0.494870
6	-5.039336	0.631092	-1.387850	7	-2.508486	-3.870809	-0.665508
6	-5.909426	1.179535	-0.455787	1	1.838948	2.328892	-1.970962
6	-5.370322	1.931904	0.572945	1	2.745832	4.622166	-2.289899
7	-4.065027	2.142473	0.720006	1	4.451163	5.478085	-0.663529
6	-0.910048	0.930991	0.007373	1	5.161596	3.983518	1.190186
7	-1.330899	-0.330934	0.013018	1	2.731253	-0.999337	2.110970
6	-0.354586	-1.233990	0.005520	1	4.556782	-2.653492	2.470463
7	0.948415	-0.967186	0.004428	1	6.391424	-2.802018	0.768330
6	1.242559	0.329759	0.004533	1	6.303405	-1.282038	-1.193749
7	0.360506	1.325032	0.000074	1	-2.383033	-1.794262	1.966500
7	2.572825	0.664520	0.013599	1	-4.697955	-2.652950	2.261699
6	3.576955	-0.314107	0.257761	1	-5.575380	-4.334247	0.621782
6	3.538132	-1.104585	1.399843	1	-4.079394	-5.070691	-1.220653
6	4.554096	-2.019870	1.591931	1	0.940481	-2.749610	-2.105624
6	5.573388	-2.102904	0.653595	1	2.554866	-4.609983	-2.468433
6	5.522961	-1.255915	-0.439758	1	2.667828	-6.447128	-0.766245
7	4.542122	-0.380070	-0.643599	1	1.153851	-6.326854	1.198698
7	-0.717059	-2.556784	-0.006515	1	-0.382759	2.986259	1.977908
6	0.240872	-3.580907	-0.251420	1	0.077841	5.418835	2.226555
7	0.287880	-4.547157	0.649845	1	-0.884528	7.002051	0.537595
6	1.142690	-5.546124	0.444630	1	-2.263175	6.062788	-1.303604
6	1.986346	-5.614511	-0.650348	1	-2.973256	0.453985	-1.964868
6	1.923098	-4.593804	-1.588720	1	-5.414550	0.045080	-2.217989
6	1.030070	-3.558582	-1.394734	1	-6.979362	1.033932	-0.527112
6	3.023419	1.992447	-0.207467	1	-6.013616	2.385441	1.320500
7	3.926556	2.433651	0.653297				

Table S3: Optimized geometry of molecule **E3**, using PBE0/Def2TZVPP method.

Atomic	Coordinates (Angstroms)			Atomic	Coordinates (Angstroms)		
Number	X	Y	Z	Number	X	Y	Z
6	3.602309	-1.438034	-1.030983	6	1.846171	4.516324	0.321364
6	2.678125	-2.404866	-0.653523	6	0.797699	3.599780	0.140470
6	3.028667	-3.764072	-0.654488	6	4.317390	4.163696	0.985704
6	4.317682	-4.163368	-0.985781	6	5.247145	3.200591	1.333683
6	5.247353	-3.200175	-1.333741	6	4.882261	1.856168	1.364497
6	4.882358	-1.855782	-1.364511	6	3.602243	1.438303	1.030994
6	1.846500	-4.516220	-0.321423	6	1.625829	5.881336	0.186603
6	0.797952	-3.599767	-0.140492	6	0.353301	6.326008	-0.123725
7	1.312493	-2.293134	-0.306983	6	-0.684600	5.411579	-0.289092
6	1.626273	-5.881253	-0.186699	6	-0.484184	4.044837	-0.159207
6	0.353786	-6.326041	0.123630	1	2.439250	6.583297	0.326055
6	-0.684190	-5.411705	0.289030	1	0.160099	7.386288	-0.230806
6	-0.483890	-4.044941	0.159182	1	-1.681185	5.769686	-0.518063
6	0.624957	-1.116411	-0.112663	1	4.582698	5.214104	0.982717
7	-0.705332	-1.180247	-0.066824	1	6.256978	3.491024	1.596125
6	-1.302559	-0.000055	0.000037	1	5.611346	1.110557	1.658155
7	-0.705402	1.180173	0.066884	1	4.583079	-5.213754	-0.982828
6	0.624889	1.116419	0.112710	1	6.257208	-3.490516	-1.596202
7	1.335454	0.000026	0.000020	1	5.611378	-1.110101	-1.658154
7	-2.691723	-0.000091	0.000035	1	2.439751	-6.583143	-0.326179
6	-3.507758	0.881642	-0.713720	1	0.160674	-7.386341	0.230681
6	-4.851735	0.556500	-0.458134	1	-1.680743	-5.769902	0.518000
6	-4.851711	-0.556791	0.458183	1	-6.907426	1.024759	-0.895703
6	-3.507720	-0.881861	0.713788	1	-6.312489	2.843310	-2.465541
6	-5.868859	1.267164	-1.087149	1	-3.948640	3.353500	-2.942276
6	-5.532986	2.279209	-1.968501	1	-6.907382	-1.025160	0.895726
6	-4.193937	2.570351	-2.235076	1	-6.312370	-2.843670	2.465583
6	-3.164519	1.876457	-1.619312	1	-3.948501	-3.353725	2.942358
6	-5.868806	-1.267508	1.087188	1	3.341503	0.394434	1.067501
6	-5.532891	-2.279530	1.968550	1	-1.304666	3.357169	-0.267057
6	-4.193830	-2.570595	2.235149	1	-2.133232	2.102032	-1.847899
6	-3.164440	-1.876649	1.619396	1	-2.133144	-2.102166	1.848002
7	1.312345	2.293195	0.306992	1	3.341481	-0.394185	-1.067455
6	2.677974	2.405047	0.653513	1	-1.304430	-3.357348	0.267055
6	3.028405	3.764282	0.654436				

Table S4: Optimized geometry of molecule **H1**, using PBE0/Def2TZVPP method.

Atomic	Coordinates (Angstroms)			Atomic	Coordinates (Angstroms)		
Number	X	Y	Z	Number	X	Y	Z
1	-9.148244	-2.925590	2.277360	6	0.727460	-0.062615	-0.031537
6	-8.937907	-1.974637	1.786239	1	-8.254471	2.801488	-2.029997
1	-7.833630	-4.142894	0.565311	6	-2.790308	0.877680	-1.042225
6	-7.671666	-2.042696	0.985862	1	5.750992	3.387433	2.696630
1	-8.886181	-1.196786	2.549098	6	-1.410127	0.805989	-1.004659
1	-9.791619	-1.743654	1.142517	6	5.312789	1.655961	1.523325
6	-7.250086	-3.241613	0.415031	1	7.048517	-1.281681	1.363052
1	-7.206338	0.032384	1.247106	1	-3.278011	1.485031	-1.794645
6	-6.899620	-0.903018	0.793191	6	3.552825	-0.209584	0.040871
1	-3.492032	-1.176219	1.547188	6	1.524238	1.022166	-0.405927
6	-6.086740	-3.284729	-0.339442	6	6.064153	2.768590	1.863769
1	-1.053970	-1.298302	1.608328	1	8.238064	-3.364839	0.817265
1	-5.766693	-4.219821	-0.784364	6	6.720398	-1.889160	0.528797
6	-5.734115	-0.935493	0.027856	1	1.056727	1.949582	-0.714819
6	-2.910362	-0.624996	0.818788	7	4.954972	-0.277216	0.081688
6	-5.331944	-2.142026	-0.544032	6	2.904550	0.952776	-0.380529
6	-1.530172	-0.698239	0.842029	1	-0.840733	1.351367	-1.748164
1	-4.230429	2.590187	0.821142	6	7.388355	-3.061897	0.216693
7	-4.969673	0.233902	-0.159862	6	5.708146	0.858146	0.451219
6	-5.110040	2.638626	0.191457	6	5.622926	-1.478404	-0.228753
1	0.811728	-2.096912	0.661964	1	3.490489	1.813062	-0.678520
6	-3.564589	0.163333	-0.127764	6	6.968075	-3.852949	-0.843919
6	-5.606712	1.468839	-0.385628	6	7.215369	3.089673	1.159541
1	-4.430031	-2.179807	-1.141614	6	5.207169	-2.271805	-1.298611
1	-5.336042	4.750055	0.422122	1	7.488575	-4.772159	-1.082613
6	-5.735705	3.853361	-0.036924	1	7.795598	3.962104	1.438406
6	-6.745079	1.545508	-1.189669	6	5.871935	-3.450719	-1.595272
1	3.243954	-2.213410	0.751400	1	4.360680	-1.959108	-1.897315
6	-6.872404	3.925056	-0.830747	6	6.861298	1.192899	-0.256950
6	1.387521	-1.223737	0.378563	1	5.535799	-4.052940	-2.431221
1	-7.136080	0.645127	-1.646667	6	7.629908	2.297563	0.091007
6	-0.742730	0.015352	-0.065392	1	7.160877	0.575811	-1.096488
6	-7.372479	2.762250	-1.401425	1	9.750736	2.140744	-0.200601
1	-7.362494	4.875341	-1.002717	6	8.883980	2.619815	-0.665050
1	4.419258	1.404612	2.080707	1	9.074185	3.693821	-0.678227
6	2.767275	-1.298193	0.423736	1	8.830210	2.266744	-1.695531

Table S5: Optimized geometry of molecule **H2**, using PBE0/Def2TZVPP method.

Atomic	Coordinates (Angstroms)			Atomic	Coordinates (Angstroms)		
Number	X	Y	Z	Number	X	Y	Z
7	4.953456	0.000000	0.000012	6	6.428109	2.673934	2.107697
7	-4.953456	0.000000	-0.000012	6	-6.428121	-2.673945	2.107650
6	5.763535	-0.894096	-0.683037	6	-6.428109	2.673946	-2.107683
6	5.763531	0.894096	0.683066	6	7.772295	-2.374727	-1.865822
6	-5.763535	-0.894100	0.683032	6	7.772284	2.374728	1.865862
6	-5.763531	0.894100	-0.683062	6	-7.772295	-2.374737	1.865809
6	7.114141	-0.571280	-0.438367	6	-7.772284	2.374738	-1.865849
6	7.114139	0.571280	0.438403	1	4.370338	-2.177737	-1.725047
6	-7.114141	-0.571282	0.438364	1	4.370328	2.177737	1.725069
6	-7.114139	0.571283	-0.438400	1	-4.370338	-2.177747	1.725035
6	3.543693	0.000000	0.000009	1	-4.370328	2.177746	-1.725057
6	-3.543693	0.000000	-0.000009	1	9.163834	-1.086818	-0.858322
6	5.407448	-1.941703	-1.525103	1	9.163829	1.086820	0.858368
6	5.407439	1.941702	1.525131	1	-9.163834	-1.086823	0.858316
6	-5.407448	-1.941711	1.525093	1	-9.163829	1.086825	-0.858362
6	-5.407439	1.941711	-1.525120	1	3.382969	-2.029485	0.671670
6	0.737187	-0.000001	0.000002	1	-3.382972	2.029481	0.671663
6	-0.737187	-0.000001	-0.000002	1	3.382972	2.029485	-0.671653
6	8.121356	-1.323779	-1.036469	1	-3.382969	-2.029482	-0.671680
6	8.121350	1.323780	1.036511	1	0.929720	-2.035275	0.665962
6	-8.121356	-1.323785	1.036462	1	-0.929723	2.035271	0.665967
6	-8.121350	1.323786	-1.036503	1	0.929723	2.035274	-0.665957
6	2.841165	-1.143851	0.363977	1	-0.929720	-2.035272	-0.665972
6	-2.841166	1.143848	0.363969	1	6.176338	-3.495754	-2.767352
6	2.841166	1.143850	-0.363963	1	6.176322	3.495754	2.767383
6	-2.841165	-1.143849	-0.363983	1	-6.176338	-3.495769	2.767333
6	1.457749	-1.141854	0.355454	1	-6.176322	3.495769	-2.767364
6	-1.457751	1.141851	0.355452	1	8.544846	-2.969708	-2.337203
6	1.457751	1.141852	-0.355447	1	8.544832	2.969709	2.337246
6	-1.457749	-1.141852	-0.355459	1	-8.544846	-2.969720	2.337187
6	6.428121	-2.673934	-2.107664	1	-8.544832	2.969722	-2.337230

Table S6: Calculated energies of the lowest excited states, and their respective oscillator strengths, in the amorphous materials based on the five organic molecules investigated in this work. Calculations were carried out within the TD-DFT framework using the combination PBE0/Def2TZVPP and a state-specific solvation calculation, with the dielectric constant ε derived as explained in the main text and reported in Table 1 of main text.States in bold characters are those chosen to de fine the optical gap.

molecule/ HOMO	excitation		$E_{exc-sta}$ (eV)	oscillator strength
**E1**/ 153	excited state 1:		3.7424	0.0244
	152 → 154	0.26191		
	153 → 155	0.62491		
	excited state 2:		**3.8066**	0.1162
	153 → 154	0.68847		
	excited state 3:		3.9299	0.2194
	151 → 154	-0.15663		
	152 → 155	-0.56453		
	153 → 154	-0.24123		
	153 → 156	-0.18276		
	153 → 157	0.16339		
**E2**/ 153	excited state 1:		4.3999	0.0080
	151 → 154	-0.18803		
	152 → 155	0.11031		
	153 → 154	0.64449		
	153 → 162	0.11161		
	excited state 2:		**4.5046**	0.3541
	151 → 154	-0.23424		
	151 → 155	0.13273		
	152 → 154	0.33932		
	152 → 155	0.16045		
	153 → 155	0.47333		
	153 → 161	-0.13248		
	excited state 3:		4.5100	0.3492
	151 → 155	0.44324		
	151 → 162	-0.12205		
	152 → 154	-0.42614		
	152 → 155	-0.11647		
	153 → 155	0.21572		
	153 → 160	-0.10639		
**E3**/ 150	excited state 1:		**3.7519**	0.3006
	150 → 152	-0.68328		
	excited state 2:		4.0059	0.3419
	147 → 152	0.13458		
	149 → 152	0.60526		
	150 → 152	-0.30019		
	excited state 3:		3.6304	0.0004
	150 → 151	0.69390		
	150 → 154	-0.10024		
**H1**/ 137	excited state 1:		**3.3733**	1.1093
	136 → 144	0.10173		
	137 → 138	0.69531		
**H2**/ 127	excited state 1:		**3.6043**	0.6322
	126 → 133	0.10682		
	127 → 128	0.69694		

Table S7: Charge-Transfer parameters computed using the local density of states for the states here studied. q^{CT} is in e, μ^{CT} is in Debye and d^{CT} in Å.

Molecule	Excited State	q^{CT}	d^{CT}	μ^{CT}
**E1**				
	1	0.502	0.198	0.477
	2	0.768	1.915	7.068
	3	0.555	1.099	2.929
**E2**				
	1	0.850	2.248	9.181
	2	0.472	0.571	1.293
	3	0.468	0.361	0.811
**E3**				
	1	0.832	2.400	9.597
	2	0.626	0.905	2.721
	3	1.140	2.887	15.816
**H1**				
	1	0.572	0.396	1.088
**H2**				
	1	0.857	0.014	0.056

2 Graphical representations of several molecular orbitals of the five molecules here studied.

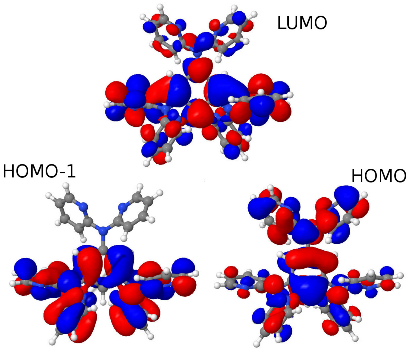

Figure S1: Isocontour plots (cutoff value of 0.018 au.) of HOMO-1, HOMO and LUMO orbitals for the molecule number **E1**, at the PBE0/Def2TZVPP level

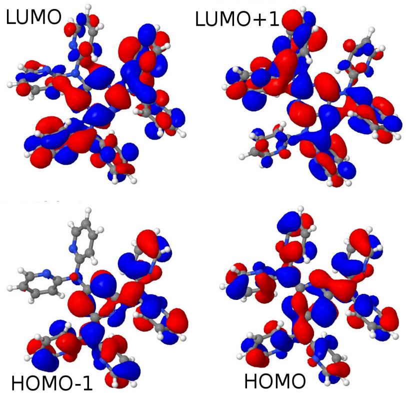

Figure S2: Isocontour plots (cutoff value of 0.018 au.) of HOMO-1. HOMO, LUMO and LUMO+1 orbitals for the molecule number **E2**, at the PBE0/Def2TZVPP level

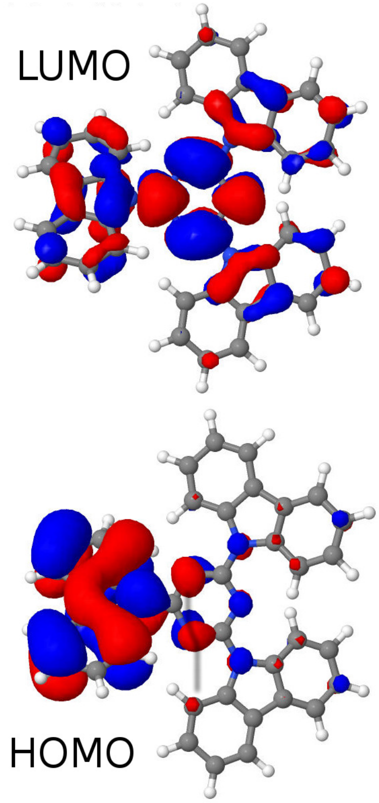

Figure S3: Isocontour plots (cutoff value of 0.018 au.) of HOMO and LUMO orbitals for the molecule number **E3**, at the PBE0/Def2TZVPP level

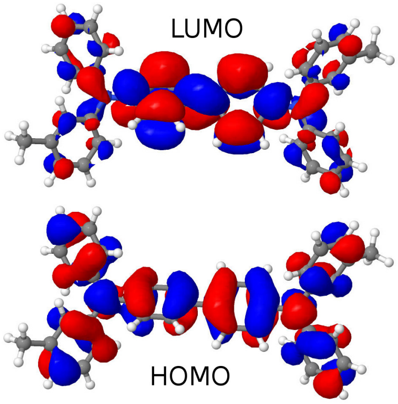

Figure S4: Isocontour plots (cutoff value of 0.018 au.) of HOMO and LUMO orbitals for the molecule number **H1**, at the PBE0/Def2TZVPP level

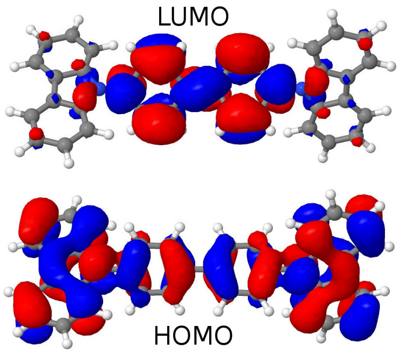

Figure S5: Isocontour plots (cutoff value of 0.018 au.) of HOMO and LUMO orbitals for the molecule number **H2**, at the PBE0/Def2TZVPP level
